# Predictors of Medical and Dental Clinic Closure by Machine Learning Methods: Cross-Sectional Study Using Empirical Data

**DOI:** 10.2196/46608

**Published:** 2024-08-30

**Authors:** Young-Taek Park, Donghan Kim, Ji Soo Jeon, Kwang Gi Kim

**Affiliations:** 1 HIRA Research Institute Health Insurance Review & Assessment Service Wonju-si Republic of Korea; 2 Center for Geospatially Enabled Society Korea Research Institute for Human Settlements Sejong-si Republic of Korea; 3 Department of Biomedical Engineering College of Medicine, Gil Medical Center Gachon University Inchon Republic of Korea; 4 KMAIN Corp Seongnam-si Republic of Korea

**Keywords:** machine learning, health facility closure, hospital closure, clinic closure, clinic bankruptcy, hospital bankruptcy, health clinic, prediction, healthcare resources, artificial intelligence, medical clinic, health insurance, health facility closure

## Abstract

**Background:**

Small clinics are important in providing health care in local communities. Accurately predicting their closure would help manage health care resource allocation. There have been few studies on the prediction of clinic closure using machine learning techniques.

**Objective:**

This study aims to test the feasibility of predicting the closure of medical and dental clinics (MCs and DCs, respectively) and investigate important factors associated with their closure using machine running techniques.

**Methods:**

The units of analysis were MCs and DCs. This study used health insurance administrative data. The participants of this study ran and closed clinics between January 1, 2020, and December 31, 2021. Using all closed clinics, closed and run clinics were selected at a ratio of 1:2 based on the locality of study participants using the propensity matching score of logistic regression. This study used 23 and 19 variables to predict the closure of MCs and DCs, respectively. Key variables were extracted using permutation importance and the sequential feature selection technique. Finally, this study used 5 and 6 variables of MCs and DCs, respectively, for model learning. Furthermore, four machine learning techniques were used: (1) logistic regression, (2) support vector machine, (3) random forest (RF), and (4) Extreme Gradient Boost. This study evaluated the modeling accuracy using the area under curve (AUC) method and presented important factors critically affecting closures. This study used SAS (version 9.4; SAS Institute Inc) and Python (version 3.7.9; Python Software Foundation).

**Results:**

The best-fit model for the closure of MCs with cross-validation was the support vector machine (AUC 0.762, 95% CI 0.746-0.777; *P*<.001) followed by RF (AUC 0.736, 95% CI 0.720-0.752; *P*<.001). The best-fit model for DCs was Extreme Gradient Boost (AUC 0.700, 95% CI 0.675-0.725; *P*<.001) followed by RF (AUC 0.687, 95% CI 0.661-0.712; *P*<.001). The most significant factor associated with the closure of MCs was years of operation, followed by population growth, population, and percentage of medical specialties. In contrast, the main factor affecting the closure of DCs was the number of patients, followed by annual variation in the number of patients, year of operation, and percentage of dental specialists.

**Conclusions:**

This study showed that machine running methods are useful tools for predicting the closure of small medical facilities with a moderate level of accuracy. Essential factors affecting medical facility closure also differed between MCs and DCs. Developing good models would prevent unnecessary medical facility closures at the national level.

## Introduction

Small medical facilities, such as hospitals and clinics, are critical in providing local community residents with health care. Approximately 6100 hospitals operate in the United States, and 35% are located in rural areas [1]. In the United Kingdom, about 1900 hospitals exist, and many small hospitals provide medical care to half the population [2,3]. Korea has 1400 small hospitals, 34,000 medical clinics (MCs), and 18,800 dental clinics (DCs) as of October 2022 [4].

The closure of small medical facilities may result in a lack of health care provision and thus bring about a sustainability issue in the community [5,6]. Small hospitals could be more critically affected than large hospitals by an equal amount of external impacts [7]. According to a study in the United States, many hospitals have closed, averaging 21 hospitals annually between 2010 and 2015 [6], and the closure of those hospitals is continuing [8,9]. In Germany, 1 small hospital closes every month [10]. In Korea, the annual closure rate of small hospitals and MCs has been reported to be 5.8% and 3.4%, respectively [11]. For these reasons, preventing the closure of medical facilities is important for the sustainability of health care systems.

Meanwhile, owing to the advancement of information technologies and statistical methods, many artificial intelligence (AI) methods, such as machine learning, have been applied to predict events in health care. They have been used for the prediction of bankruptcy [12-14], timely attendance and no-shows in medical appointments [15,16], hospital admissions and discharges, and hospital length of stay [17-20]. Although there are several studies on hospital or clinic closure [21-24], no study predicts the closure of MCs or DCs using AI methods and empirical data.

Regarding this study on the closure of hospitals or clinics using machine learning methods, there was 1 case where machine learning methods were used to study the closure of small hospitals. A study conducted in the United States investigated the predictive factors related to hospital bankruptcy using AI methods and found that various factors such as facility age, market concentration, and Medicare percentage were associated with hospital bankruptcy. This study used AI techniques such as a linear support vector machine (SVM) model with a hinge function, a perceptron neural network model, and so on [24]. A recent study predicted an annual number of hospital patients using several machine learning techniques and found that the most important factor associated with a predicted number of inpatients was the number of beds, followed by the number of nurses; in contrast, the best predictive factor for the outpatient was several doctors followed by several local households [25]. When asked about the important factors for an annual number of hospital patients, most people would say “physicians” or “nurses.” However, machine learning suggests that the number of beds and nurses is important in the inpatient section because the number of patients is produced through beds and by nurses. In contrast, the number of doctors is important in the outpatient section because they sequentially care about patients. Machine learning techniques also pointed out that the number of households in local areas is important for predicting annual outpatients.

Various factors have been known to be associated with the closure of medical facilities, such as affiliation status and financial distress [22,26,27], profitability [28], size of hospital and locality [29], and market competition [30]. However, they used traditional statistical methods such as logistic regression (LR) rather than machine learning. We can develop a good model that predicts the closure of hospitals and MCs with advanced technologies. In that case, we can help health care policy makers improve the sustainability of health care systems and maintain healthy local medical communities by targeting those factors with appropriate policies.

This study is important from several perspectives. This study is the first to use machine learning to predict the closure of MCs and DCs and study closure-related factors. The results expand the scope of business closure research from current hospitals to clinics, DCs, pharmacies, nursing homes, and long-term care hospitals. Second, this study will be of particular interest to those who want to start a new business in the health care field. In many countries, every year, many medical professionals and pharmacists graduate from universities or colleges and strive to open their clinics. This study provides support for using AI to identify suitable areas for clinics and pharmacies in their business plan. Finally, the results obtained from this study help to fill the research gap between good prediction and reality, which will contribute to developing good methodological models predicting the closure of health care institutions.

Therefore, this study aimed to predict the closure of MCs and DCs, including selecting the best model and identifying significant factors critically affecting this process. The organizational characteristics and knowledge gained from this study could provide health care policy makers and international colleagues with useful information to build sustainable and healthy health care delivery systems.

## Methods

### Study Setting

This study is conducted in Korea, as it has many medical facilities, and closures occur frequently. Therefore, using AI methods, Korea serves as a good experimental setting to observe and predict the closure of medical facilities. Institutionally, Korea has adopted the national health insurance system. For the same types of medical care, each medical facility receives an equal amount of reimbursement because of the setting of the national health insurance systems. There are also many private and public medical facilities, such as small hospitals, MCs, and DCs. Patients can choose any primary care clinic without constriction. Judging from these facts, the market is very competitive for survival, and dynamic factors may affect the closure of medical facilities. This study targeted MCs and DCs as study participants because there are many clinics and frequent occurrences of events such as clinic closures. Approximately 30,000 MCs and 25,000 DCs are operating as of December 31, 2022. Owing to the high competition, there are several closures.

Regarding the recruitment settings and procedures of study participants, this study used a research data set from the Health Insurance Review and Assessment Service (HIRA). The HIRA is a third-party administrator providing professional medical claim review and assessment services to Korea’s National Health Insurance Service. Every health care organization, such as hospitals and clinics, should register or report their openness and closure of facility to the government through a Unified Portal Reporting Healthcare Resource [31]. This portal is run by the HIRA, which collects data for administrative work. In detail, this study took data from the HIRA on clinics that operated and closed for 2 years from 2020 to 2021. Then, the participants for this study were selected from operating and closed clinics based on region at a ratio of 2:1. The detailed method will be explained in the Study Design section. The second data set was from an open public data set on residential and household information [32] run by the Ministry of Interior and Safety. This study collected data such as area code, local population, population growth, and number of households from the Ministry of Interior and Safety. These data were analyzed by linking them with the region code to which the clinics belong. The last one was from information on subway stations provided by the National Geographic Information Institute [33]. Using the clinic’s location information, we identified whether there was a subway station within a 1 km radius or not. National Geographic Information Institute provided this information.

### Study Design

This study adopted a cross-sectional study design for over a 2-year observation period. The unit of analysis was individual MCs and DCs. For this study, the closure status of MCs and DCs during the period 2020-2021 was identified. By observing 2 time points on December 31, this study identified whether the clinics closed or not and coded them as a group of clinics closed (1) or not (0). Using the closed group, we selected the participants for this study from closed and operating medical facilities with a ratio of 1:2 in MCs and DCs, respectively, because the proportion of closed facilities was too low, constituting less than 5% of the total study population. Training and testing participants were processed with a 4:1 ratio and 5-fold cross-validations for 4 machine learning algorithms: LR, SVM, random forest (RF), and Extreme Gradient Boost (XGB) methods.

**Figure 1:**
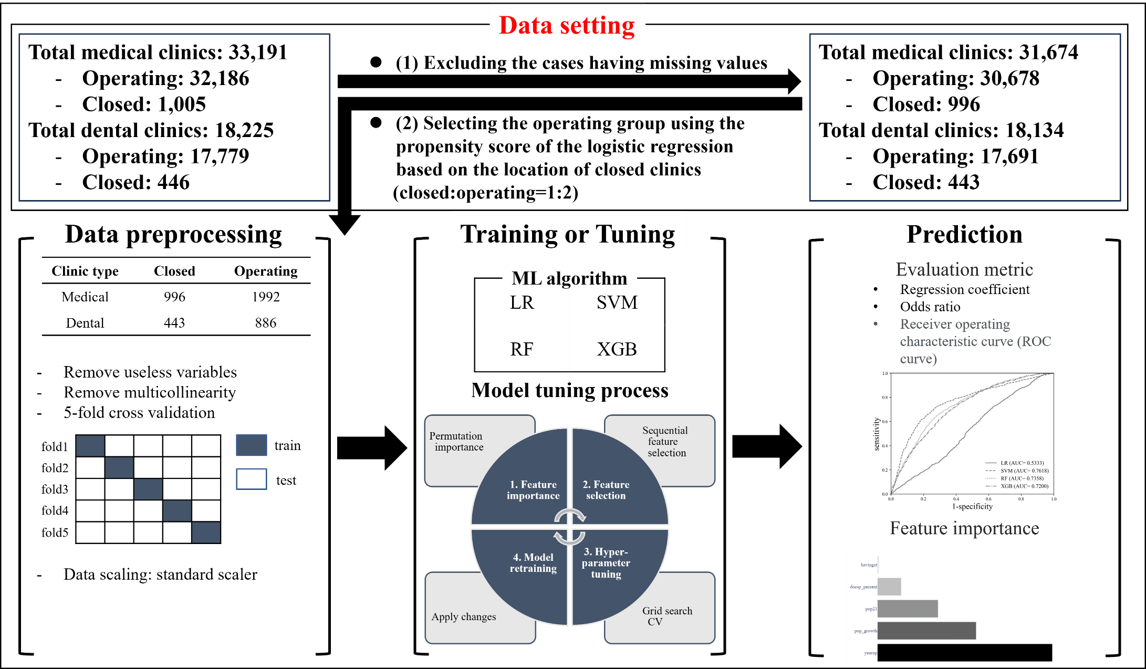
Overall diagram for data processing steps: a cross-sectional study design over 2 years (2020-2021). AUC: area under the curve; CV: cross-validation; LR: logistic regression; RF: random forest; ROC: receiver operating characteristic; SVM: support vector machine; XGB: Extreme Gradient Boost.

The amount of data was sufficient, so the model learning the characteristics of each variable would be suitable. Therefore, we used stratified cross-validation to maintain the same ratio of running and closed clinics for each fold to prevent bias instead of using simple K-fold cross-validation. This study selected the LR and SVM methods because the former is the most classical model among the various machine learning models, and the latter is considered the fundamental model of deep learning and performs well in dealing with complicated models. This study also selected the RF and XGB methods because they are most frequently used for machine learning, with moderate modeling performance. Using these methods, this study predicted the probability of closure of MCs and DCs. After developing the prediction model, we evaluated the prediction results. The results were expressed through cross-validation. Figure 1 demonstrates the data processing flow or diagram of this study.

### Outcome and Covariate Measures

This study predicted the closure or nonclosure of MCs and DCs, the major outcome variable. The other input variables were chosen based on previous studies but within the boundary of data availability: years of operation, number of patients, annual variation in the number of patients, having beds or not (for clinics), having computed tomography devices or not (for clinics), having magnetic resonance imaging devices or not (for clinics), number of physicians (for clinics) or dentists (for DCs), percentage of medical or dental specialists, percentage of nurses or hygienists, the local population of the year 2021, population growth rate (%), number of clinics or DCs within the local area, and having a subway station or not within 1 km.

The input variables were selected based on several previous studies. These studies used general statistical methods such as LR rather than machine learning. A study found that the closure of MCs was associated with years of operation, for-profit status, the proportion of specialists among all physicians, the number of clinics within the local area, the closure of DCs related to the proportion of specialized dentists among all dentists, the proportion of nurse and dental hygienists among all nursing staff, and several DCs within the local area [11]. Several studies on the closure of hospitals and business firms presented that closure was associated with factors such as facility age, financial status, affiliation status, and market factors [22,24,26,27]. This study used all available variables as input variables.

The annual number of patients was observed in 2020 for operating MCs and DCs. For closed MCs and DCs, the number of patients a year before closure was used. For the annual variation in the number of patients, the SD of the number of patients for 5 years was calculated. If clinics had less than 5 years of operation, we calculated the SD of the number of patients for the years of operation. The percentage of medical or dental specialists was calculated by (specialized physicians for clinics (dentists for DCs)/sum of physicians of clinics (dentists for DCs) × 100), and the percentage of nurses for MCs was calculated by (number of nurses/(number of nurses + nurse aids)× 100). The percentage of nurses for DCs was calculated by ((number of nurses + number of dental hygiene)/(number of nurses + number of dental hygiene + nurse aids)) × 100. Local area means an administrative district in Korea.

### Statistical Analysis

Various machine learning techniques are used to predict patients’ clinical behavior and decision-making [34,35]. This study used similar methods to those in previous studies. Before the main analysis, we took the following data preprocessing steps. First, by using the SAS program, we excluded all missing data. Second, as mentioned above, this study selected study participants from closed and running medical facilities with a ratio of 1:2 because the proportion of event groups, meaning closed facilities, was too low, being less than 5%. This process was conducted in MCs and DCs, respectively. This process was conducted with the SAS program, and this study used the propensity matching score. Third, for the cases of numeric valued data, this study rescaled them to have a normal distribution with a mean of 0 (SD 0) and a variance of 1, which is called the standard scaling process.

This study used MedCalc (version 14.8.1; MedCalc Software Ltd) to reanalyze the predicted results regarding the machine learning module. When we calculated the receiver operating characteristic curve comparison, Stata (version 11; StataCorp LLC) was used. Regarding the machine learning algorithm, this study used the LR, SVM, and RF, which are provided by the library of scikit-learn (version 0.23.2; Python Software Foundation) and xgboost (version 1.4.0; Python Software Foundation). Regarding feature selection, this study used an algorithm that shows the permutation importance, which the library of Eli5 provides for computing feature importance. This study used 23 and 19 variables to predict the closure of MCs and DCs, respectively. Key variables were extracted using the permutation importance from those variables. The sequential feature selection technique was also used. Finally, this study selected 5 and 6 variables of MCs and DCs, respectively, for model learning.

Regarding the validation methods of the models, this study used sensitivity and specificity indices following the definition of a previous study [36]. Regarding the prediction capacity of the models, this study used area under the curve (AUC), which was calculated based on sensitivity and specificity. According to the *Standards for Reporting Diagnostic Accuracy Studies 2015* [37], it is suggested that specificity, sensitivity, and receiver operating characteristic curves be used for diagnostic evaluation. Thus, this study chose them and presented its study outputs. This study used Python (version 3.7.9; Python Software Foundation) to solve the main analysis.

Regarding AI methods and paper writing, this study discloses that no generative AI was used at any stage of writing this paper. Finally, the computer operating and hardware system were Intel Core i9-10900 CPU @ 2.80 GHz and Windows 10 OS.

### Ethical Considerations

This study was approved by the institutional review board (IRB) of the HIRA on February 10, 2022 in Korea (2022-010-001). The IRB letter says that it is exempted from proceeding with the formal IRB review due to the use of the secondary health insurance administrative data. The letter also says that it has nothing to do with obtaining informed consent from research participants because the study participants are individual clinics.

## Results

### General Characteristics of the Study Participants

Table 1 presents the basic characteristics of clinics and DCs regarding closure status. Closed MCs are more likely to have longer operating years, fewer patients, and less variation in the number of annual patients compared to those operating. Closed DCs are less likely to be specialized and have fewer specialized nurses compared to the others.

**Table 1:** General characteristics of the study participants (medical and dental clinics) by clinic closure status.

Variables			Medical clinics											Dental clinics							
			Closure		Nonclosure		All		*P* value			Closure				Nonclosure		All		*P* value	
Study participants, n			996		1992		2988		—^a^			443				886		1329		—	
Operation (years), mean (SD)			15.6 (11.7)		13.4 (8.4)		14.1 (9.7)		*.001* ^b^			15.7 (11.8)				14.6 (9.2)		14.9 (10.2)		*.08*	
Patients, mean (SD)			12,028.6 (13,157.4)		14,465.3 (11,557.9)		13,653 (12,166.8)		*.001*			1178.3 (1164.7)				1518.9 (1162.1)		1405.4 (1173.6)		*.001*	
Annual variation of the number of patients, mean (SD)			3104.1 (3715.7)		2665.6 (3670.8)		2957.9 (3706)		*.002*			260.9 (396.2)				228.6 (311.5)		239.4 (342.3)		*.10*	
**Having beds, n (%)**										*.50*											—
	Yes	158.364 (15.9)		334.656 (16.8)		493.02 (16.5)					—				—		—		—		
	No	837.636 (84.1)		1657.344 (83.2)		2494.98 (83.5)					—				—		—		—		
**Having computed tomography, n (%)**										*.35*											—
	Yes	12.948 (1.3)		35.856 (1.8)		47.808 (1.6)					—				—		—		—		
	No	983.052 (98.7)		1956.144 (98.2)		2940.192 (98.4)					—				—		—		—		
**Having magnetic resonance imaging, n (%)**										*.25*											—
	Yes	3.984 (0.4)		15.936 (0.8)		17.928 (0.6)					—				—		—		—		
	No	992.016 (99.6)		1976.064 (99.2)		2970.072 (99.4)					—				—		—		—		
Doctors or dentists, mean (SD)			1.3 (1.2)		1.4 (1.1)		1.4 (1.2)		*.43*			1.4 (0.8)				1.5 (0.9)		1.5 (0.9)		*.09*	
Medical or dental specialists, mean% (SD)			87.9 (31)		88.7 (29.8)		88.4 (30.2)		*.48*			10.5 (26.6)				14 (28.2)		12.8 (27.7)		*.03*	
Nurse or hygienist specialized, mean% (SD)			9.6 (24)		10 (23.3)		9.9 (23.5)		*.65*			54.9 (40.8)				60.1 (37.6)		58.4 (38.8)		*.02*	
Local population, mean (SD)			353,320 (179,791.1)		353,312.3 (179,759.9)		353,314.9 (179,740.2)		*.99*			360,496.1 (172,732.1)				360,496.1 (172,634.5)		360,496.1 (172,602)		*.99*	
Population growth rate, mean (SD)			–0.112 (11)		–0.120 (11)		–0.117 (11)		*.98*			–1.299 (10.3)				–1.299 (10.3)		–1.299 (10.3)		*.99*	
Clinics or dental clinics within a local area, mean (SD)			376.4 (471.2)		377.1 (471.3)		376.9 (471.2)		*.96*			193.2 (148.7)				191.7 (145.6)		192.2 (146.6)		*.85*	
**Subway station within 1 km, n (%)**										*.58*											*.15*
	Yes	445.212 (44.7)		868.512 (43.6)		1314.72 (44)					237.005 (53.5)				436.798 (49.3)		673.803 (50.7)				
	No	550.788 (55.3)		1123.488 (56.4)		1673.28 (56)					205.995 (46.5)				449.202 (50.7)		655.197 (49.3)				

^a^Not applicable.

^b^Italicized values indicate the *P* value of *t* test for numerical variables, or the *P* value of chi-square test for categorical variables.

### Model Performance Using Cross-Validation

**Figure 2:**
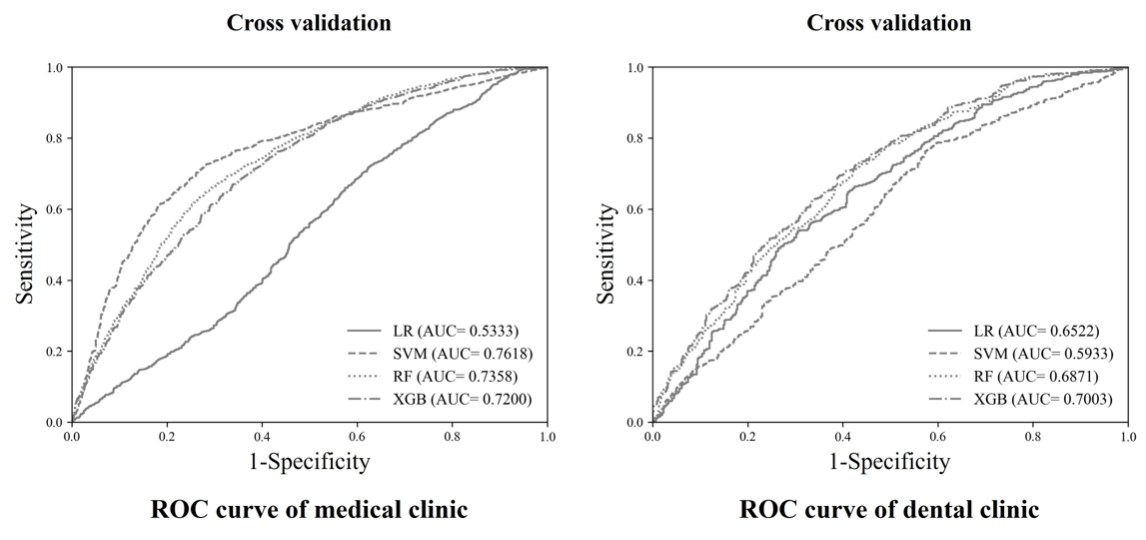
Predicted model performance for medical and dental clinics. The ROC represents the receiver operating characteristic plotting the true positive rate (sensitivity) against the false positive rate (1-specificity) for different classification thresholds. AUC: area under the curve; LR: logistic regression; RF: random forest; ROC: receiver operating characteristic; SVM: support vector machine; XGB: Extreme Gradient Boost.

Figure 2 presents the model performance results of 4 models in MCs and DCs. In MCs, model performance was highest in SVM for the AUC index, followed by RF, XGB, and LR. In DCs, model performance was highest in XGB, followed by RF, LR, and SVM.

### Model Cross-Validation

Table 2 presents the performance indicator for each model. In the cross-validation, the AUC of MCs was highest in SVM (0.762), followed by RF (0.736). The AUC of DCs was highest in XGB (0.700), followed by RF (0.687).

**Table 2:** Performance indicator for each model (cross-validation) by medical and dental clinics.

Clinic types and models			Sensitivity, AUC^a^ (95% CI)		Specificity, AUC (95% CI)		AUC (95% CI)		*P* value (AUC)
**Medical clinics**									
	LR^b^	0.996 (0.992-0.998)		0.033 (0.023-0.046)		0.533 (0.515-0.551)		.004	
	SVM^c^	0.896 (0.881-0.909)		0.326 (0.297-0.356)		0.762 (0.746-0.777)		<.001	
	RF^d^	0.939 (0.928-0.949)		0.278 (0.250-0.307)		0.736 (0.720-0.752)		<.001	
	XGB^e^	0.823 (0.805-0.839)		0.480 (0.448-0.511)		0.720 (0.704-0.736)		<.001	
**Dental clinics**									
	LR	0.990 (0.981-0.995)		0.034 (0.019-0.055)		0.652 (0.626-0.678)		<.001	
	SVM	0.998 (0.992-0.999)		0.005 (0.001-0.016)		0.593 (0.566-0.620)		<.001	
	RF	0.916 (0.896-0.934)		0.278 (0.236-0.322)		0.687 (0.661-0.712)		<.001	
	XGB	0.903 (0.882-0.922)		0.323 (0.279-0.369)		0.700 (0.675-0.725)		<.001	

^a^AUC: area under the curve.

^b^LR: logistic regression.

^c^SVM: support vector machine.

^d^RF: random forest.

^e^XGB: Extreme Gradient Boost.

### Major Factors Affecting Closure by Clinic Types

Years of operation, population growth, number of residents within the local area, and percentage of medical specialists are crucial factors for closing MCs. In contrast, number of patients, annual variation in the number of patients, years of operation, percentage of dental specialists, and number of residents within the local area are considered essential factors affecting DC closures.

Table 3 presents the crucial factors measured by permutation importance across 4 models. The year of operation for MCs had the highest score (0.194), followed by population growth (0.110). For DCs, the number of patients had the highest score (0.096), followed by annual variation in the number of patients (0.060).

**Table 3:** Selecting important factors based on the mean score of the permutation feature importance by clinic types (medical and dental clinics) and prediction models.

Clinic type and factors			LR^a^		SVM^b^		RF^c^		XGB^d^		Mean score (SD)
**Medical clinics**											
	Having CT^e^	0.001		–0.004		0.005		0.003		0.001 (0.001)	
	Medical specialty (%)	0.004		0.046		0.034		0.021		0.026 (0.002)	
	Local population	–0.009		0.150		0.073		0.055		0.067 (0.004)	
	Population growth	–0.004		0.224		0.116		0.103		0.110 (0.004)	
	Operation (years)	0.043		0.284		0.244		0.207		0.194 (0.01)	
**Dental clinics**											
	Dentists (n)	–0.001		0		0.002		0		0 (0.009)	
	Local population	0		0.003		0.004		0.004		0.003 (0.004)	
	Dental specialty (%)	0.018		0.006		0.008		0.005		0.009 (0.004)	
	Operation (years)	–0.003		0.018		0.059		0.065		0.035 (0.015)	
	Annual variation in the number of patients (n)	0.056		0.014		0.078		0.094		0.060 (0.012)	
	Patients (n)	0.135		0.033		0.111		0.106		0.096 (0.01)	

^a^LR: logistic regression.

^b^SVM: support vector machine.

^c^RF: random forest.

^d^XGB: Extreme Gradient Boost.

^e^CT: computed tomography.

## Discussion

### Principal Results

This study found that it is feasible to predict the closure of MCs and DCs using machine learning methods with a fair level of the validation score measured by the AUC. Interestingly, MCs and DCs have different best-fitting models. The best-fitting MC and DC closure models were in SVM and XGB, respectively. Finally, except for years of operation, different important features affected the closure of MCs and DCs. Years of operation were an important feature affecting the closure of both MCs and DCs. External environmental factors, such as population growth and local residential population, largely influenced the closure of clinics. However, the closure of DCs was influenced by the internal characteristics of the facility, such as the number of patients and annual variation in the number of patients.

### Comparison With Prior Work

This study indicates the feasibility of predicting clinic closure with machine learning methods. This result aligns with previous studies in which many AI methods well predicted patients’ clinical behaviors [34,35] and partially predicted hospital closure and general firm bankruptcy [12-14,24]. Other than that, we could not find any similar previous studies targeting MCs and DCs; thus, it was impossible to compare the results of this study with others. However, our study results suggest that the same machine learning technologies could be applied to predicting the closure of medical facilities, such as many long-term care facilities, pharmacies, and clinics with oriental medicine.

Regarding the model performance to predict closure, SVM and XGB were the best-fitting models to predict the closure of MCs and DCs, respectively. A very interesting finding is that each study group has its best-fitting model. While a study on bankruptcy prediction presented that XGB is the best performer, the best-fit model for predicting the annual number of inpatients and outpatients was RF and linear regressor, respectively [25,38]. These results suggest that future research should refer to the optimal models of previous studies and include such models as alternatives in their research methods.

For cross-validation using the AUC score, MCs had the highest score in SVM (0.762) followed by RF (0.736) and DCs had the highest score in XGB (0.700) followed by RF (0.687). These study results suggest 2 things. The first is about the models’ cross-validation per se. Generally, an AUC score above 0.70 would be considered “fair” for validating the model [39]. Several previous studies dealing with various health care participants have a broad range of AUC and modeling accuracy ranging from 0.69 [24] to 0.85 AUC [40]. Compared to these previous validation scores, this study has a fair cross-validation score, and thus, there is ample potential to develop further good models based on this study. Second, this study suggests that the model performance of MCs and DCs needs to improve further. As evidenced by the results of this study, the overall cross-validation score of each model in MCs is slightly higher than that of DCs. Further search and analysis are necessary to investigate why these are different. This study suggests that methodological approaches that involve selecting different input variables by each model with their own are necessary.

This study found that years of operation were an important factor affecting the closure of MCs and DCs. Clinics performing medical practices over a longer period in 1 place might consider moving to other places, which is why factors such as years of operation are significant factors affecting clinic closure. Our study results showed that external environmental factors, such as population growth and local residential population, are essential to consider in the closure of clinics, and the internal characteristics of DCs, such as the number of patients and annual variation in the number of patients, critically affect the closure of DCs. The results of this study partially align with those of a previous study in which factors such as medical and nurse specialties (both in MCs and DCs), year of operation (in both), and market competition are closely associated with the closure of medical facilities [11]. Nevertheless, there are some differences in that while the previous study was based only on LR investigating factors associated with the closure of MCs, this study involved predicting and identifying critical factors affecting the closure of clinics with various AI methods.

These research results tell people who want to open a new MC or DC what factors are related to business closure. This study argues that these factors must be considered in countries where many MCs and DCs are operating. This study used several machine learning methods, but they needed more accuracy. This study presents the challenge of increasing the accuracy of the methodology, thus providing fundamental reference values for these analysis methods.

Regarding the results of this study concerning hospital closure, there are distinctive differences between hospitals and clinics. While factors such as affiliation status, financial distress, size of the hospital, locality, low-profit margin, and market competition were critical factors affecting hospital closure [22,26,27,29,30], features such as years of operation, local population, and number of patients were significant factors affecting MC and DC closure [11]. However, direct comparisons between hospitals and clinics are still unreasonable because the participants of this study are different.

This study is valuable in several respects. First, this study is the first to predict the closure of MCs and DCs. There have been several studies on the closure of hospitals but not clinics. Second, studies on clinic closure are very important because clinics play a dominant role in providing health care to the community residents. Identifying closure factors is crucial to prevent the breakdown of the health care delivery systems of local communities. By investigating their survival through this kind of study, we can obtain useful information to increase the sustainability of their business survival. Third, this study had highly valid data sets comprising national data. A study using a large cumulative data set would be invaluable because the validity of study results increases as the sample size of a study increases.

### Limitations

This study has several limitations. First, although this study was based on a 2-year national data set, few clinics have closed. This study conducted predictions using 2-year aggregated data by selecting operating clinics and matching them with closed clinics. This process might lead to the loss of some valuable information. However, this study had a rigorous methodology in that it increased comparability by equally weighting the risk factors to those study participants within the same locality. Second, although financial factors are critical factors affecting hospital closure [22,27], this study did not include that variable. However, to minimize its impact, this study included the number of patients as a potential proxy variable of financial factors. Third, this study design is of closed clinics, and running clinics were selected at a ratio of 1:2 based on the locality of study participants using the propensity matching score of LR, which might result in an under-sampling issue and, thus, would have the risk of overfitting. We suggest that future research could consider a hypothesis-free approach, such as repeated undersampling, to overcome this study’s limitations. Fourth, the data collection period is within the COVID-19 pandemic. The COVID-19 pandemic is very special, in which the closure of small clinics might be common. Small health care institutions are more greatly affected by external forces [7]. The data from the pandemic period would differ from those we see today. Thus, the interpretation of study results needs consideration of this background. However, although data were collected during this unique period, this study argues that the impact of COVID-19 would equally affect those clinics running and closed. This may minimize its negative impact on study data. Finally, the results of this study may be limited to Korea and countries with health care delivery systems similar to those in Korea. Suppose the operation or closure of MCs is dependent on governmental plans. In that case, the results of this study may not apply to those countries because this study is based on free business medical markets.

### Conclusions

This study predicted the closure status of MCs and DCs and found that composing models that predict the closure status of clinics is feasible. Among the 4 models, SVM was the best-fit model for MCs and XGB for DCs. Years of operation were an important factor that critically affected the closure of MCs and DCs. Additionally, environmental factors critically affected the closure of MCs; in contrast, DC’s internal features, such as the number of patients, were critical factors affecting the closure of DCs. These discoveries increase the range of our knowledge and understanding that machine learning techniques could be useful in predicting the closure of small medical institutions and applicable to long-term care hospitals, nursing homes, and pharmacies. This study also indirectly suggests that governments could conserve health care resources and prevent wastage if they develop good prediction models and provide information to medical or potential medical providers. We hope this study will inspire similar studies with further dynamic methods, leading to knowledge expansion and providing various insights to policy makers and researchers in related fields.

## Abbreviations

AI: artificial intelligence
AUC: area under the curve
DC: dental clinic
HIRA: Health Insurance Review and Assessment Service
IRB: institutional review board
LR: logistic regression
MC: medical clinic
RF: random forest
SVM: support vector machine
XGB: Extreme Gradient Boost

## Data Availability

The data sets used and analyzed during this study are available from the corresponding author upon reasonable request. In addition, the data sets on clinic closures may be obtained from the HIRA by reasonable request.
